# Identification of Circulating *hsa-miR-324-3p* and *hsa-miR-331-3p* Exchanges in The Serum of Alzheimer’s Patients and Insights into The Pathophysiological Pathways

**DOI:** 10.22074/cellj.2021.7047

**Published:** 2021-05-26

**Authors:** Maryam Heydari, Zohreh Hojati, Moein Dehbashi

**Affiliations:** Division of Genetics, Department of Cell and Molecular Biology and Microbiology, Faculty of Biological Science and Technology, University of Isfahan, Isfahan, Iran

**Keywords:** Alzheimer’s Disease, MicroRNAs, Quantitative Reverse Transcription Polymerase Chain Reaction, Serum

## Abstract

**Objective:**

Alzheimer’s disease (AD) is a type of dementia. Currently, there are not any existing and reliable methods for the prognosis or diagnosis of AD. Hence, finding a diagnostic/prognostic biomarker for AD helps physicians to prescribe the treatments and methods preventing disease progression. Circulating microRNAs (miRNAs) are the most promising biomarkers due to their non-invasive and easily accessible for diagnosis and prognosis of AD. The aim of current study is to evaluate expression levels of two unwell-known circulating miRNAs including *hsa-miR-324-3p* and *hsa-miR-331-3p* in serums of AD patients and to understand their roles in AD physiopathogenesis by in silico analysis.

**Materials and Methods:**

In this case and control study, to get the gene targets related to these two miRNAs, TargetScan, miRTargetLink Human and mirDIP web servers were applied. In addition, gene networks and gene ontology enrichment analysis were performed by STRING 10.5, KEGG and ShinyGO v0.41. Experimentally, expression levels of these two miRNAs in the serum of 21 patients with AD and 23 healthy individuals were compared using the quantitative reverse transcription polymerase chain reaction (qRT-PCR) method.

**Results:**

The pathophysiological pathways associated with these two miRNAs were nucleotide metabolism and cellular response to stress pathway. Furthermore, the upregulated expression levels of *hsa-miR-324-3p* and *hsa-miR-331-3p* in comparison with the healthy control serums were not statistically significant (P>0.05).

**Conclusion:**

Non-significant results were obtained from the expression levels of AD patients and two significant pathways were obtained by networks and gene enrichment analysis.

## Introduction

Alzheimer’s disease (AD) is a neurodegenerative and age-dependent disease in which the patients suffer loss of memory, cognitive and behavior dysfunctions (1-3). Investigations on postmortem AD brains showed that is mainly relied on intracellular neurofibrillary tangles (NFTs), extracellular (Amyloid β) Aβ plaques, synaptic damage, loss of synapses, loss of synaptic proteins, proliferation of reactive astrocytes and activated microglia, deficiency in cholinergic neurons, an age-dependent imbalance in hormones, as well as structural and functional alterations in mitochondria (4-13). Early manifestations in the pathogenesis and progression of AD include synaptic damage, loss of synapses and mitochondrial oxidative damage (12). Besides, cognitive decline in AD patients are obtained from lack of synapses and synaptic damage, as the most obvious features (14). Aging is a step, making the risk factor for developing AD in the society. However, prognosis and diagnosis of AD can help physicians recognize this neurological disorder to prescribe the drugs delaying or preventing disease progression. 

In this way, the molecular biomarkers are under the spotlight for their potential roles. Nowadays, recent achievements demonstrated that circulating and blood-based miRNAs, as small non-coding RNAs (20-24 nucleotides), can be applied as early detectable peripheral biomarkers for aging and AD as well as the other neurological diseases (15, 16). There are some miRNAs that are involved in most of the neurodegenerative diseases (8, 17). Kumar et al. (18) demonstrated the discovery and validation of the unique circulating miRNA signatures including hsa-let-7d-5p, hsa-let-7g-5p, hsa-miR-15b-5p, hsa-miR-142-3p, hsa-miR-191-5p, hsa-miR-301a-3p and hsa-miR-545-3p in plasma, which could identify AD patients from healthy controls.

Some studies also showed miR-324-3p was downregulated in the brain tumor cells and suggested its hypothetical role as tumor suppressor (19-21). Liu et al. (22) showed that miR-324-3p was downregulated in the brain of an embolic stroke model and its expression may be an indicator of recovery. In addition, Stappert et al. (21) reported that miR-324-3p was upregulated in neural cells compared to human embryonic stem cells and it was further enhanced upon differentiation. However, Vallelunga et al. (23) observed that miR-324-3p, as a circulating miRNA, was upregulated in the serum of Parkinson’s disease (PD) and Multiple System Atrophy (MSA) patients in comparison with healthy individuals. Until now, the literature on the role of miR-324-3p in the function and mechanism of AD is undefined. This clue persuaded us to find the miR-324-3p expression level in the serum of AD patients and the signaling pathways in which this miRNA can be computationally involved. On the other hand, Wang et al. (24) reported miR-331-3p was downregulated in the cerebral cortex of Alzheimer’s patients (24). Olivieri et al. (25) showed the upregulation of miR-331-3p in the plasma of elderly individuals. Balakathiresan et al. (26) used a rat model of learned helplessness stress to identify significantly modulated miRNAs in serum after traumatic stress and reported miR-331-3p was upregulated. Epis et al. (27) showed HuR and miR-331-3p participate in the overexpression of ERBB-2 observing in some prostate cancers. Saba and Booth (28) analyzed miRNA expression in the mouse brain during prion-induced neurodegeneration and reported that miR-331-3p was up-regulated. Wang et al. (24) showed downregulation of **hsa-miR-331-3p** in the brain white and gray matter of the female AD patients. Zanette et al. (29) showed the upregulation of *hsa-miR-331-3p* in acute lymphoblastic leukemia (ALL) malignancies. This clue motivated us to focus on the role of *hsa-miR-331-3p* in AD patients as a circulating miRNA and to find computationally the disease related pathways. Therefore, two miRNAs in this paper, including hsa-miR-324-3p and hsa-miR-331-3p, were considered by their expression changes and enrichment analyses in serum of AD patients based on pathophysiological approach.

## Materials and Methods

### Network and enrichment analysis

The publicly available databases including TargetScan (http://www.targetscan.org/vert_71), miRTargetLink Human (https://ccb-web.cs.uni-saarland.de/mirtargetlink) and mirDIP (http://ophid.utoronto.ca/mirDIP/index.jsp) were applied. The targets of hsa-miR-324-3p and hsa-miR-331-3p were obtained using the options including strong evidence, weaker evidence and predicted interactions from miRTargetLink. In addition, the targets of these two miRNAs were achieved according to the score class (very high, high and medium) from mirDIP. Furthermore, STRING 10.5 (https://string-db.org), KEGG biological pathway (https://www.genome.jp) and ShinyGO v0.41 (Gene Ontology Enrichment Analysis + more; http:// bioinformatics.sdstate.edu/go) by P value cut off= 0.05 for false discovery rate (FDR) were utilized to determine the gene networks and gene ontology enrichment analysis.

### Ethics statements

This research was done in accordance with the Declaration of Helsinki. Informed consents were obtained from all individual participants/their families for this research. In addition, the research was confirmed by the Ethics Committee of the University of Isfahan (Isfahan, Iran), with the approval code of 98/50297.

### Serum samples

In this case and control study, the patients included in this survey were people with AD residing at the Sadeghyeh Welfare Organization (Isfahan, Iran) between December 2016 and February 2017. For this aim, 44 blood samples, including 21 patients with AD and 23 healthy individuals were collected. The AD patients were diagnosed following the NINDS-ADRDA criteria (30) and revised criteria from the National Institute on Aging-Alzheimer Association (31). Blood samples were gained by venous puncture, permitted to be clotted for 30 minutes and centrifuged at 2000 rpm for 10 minutes to get the sera sample. The sera were then collected and allocated into the new tubes and stored at -80˚C until. 

### RNA isolation

All RNAs (including miRNA) were isolated by miRCURY™ RNA Isolation Kit- Biofluids (Exiqon, Denmark) from serum samples according to manufacturer’s instruction. The ratio between the 260 nm and 280 nm absorbance (A260/A280) provided us with an estimate of purity of the RNA. The purity of extracted RNAs was analyzed by NanoDrop Spectrophotometer (ND-1000, Thermo Fisher, USA). All purified RNAs had an A260/A280 ratio of 1.8-2.1 in 10 mM Tris-Cl, pH=7.5. *hsa-miR-451* (32, 33) and *UniSp6* (recommended by kit) were used as internal control and the spike-in control, respectively. *UniSp6* spike-in control was added to the RT reaction mix.

### cDNA synthesis, quantitative reverse transcription polymerase chain reaction and polyacrylamide gel electrophoresis 

cDNAs synthesis for *hsa-miR-324-3p*, *hsa-miR-331- 3p* and *hsa-miR-451* (internal control) were performed by miRCURY LNA™ Universal RT microRNA PCR (Exiqon, Denmark), as indicated by the manufacturer, and *UniSp6*, RNA Spike-in template was used as a positive control. cDNA products were incorporated into a master mix composed of 10 pmol/μl of *hsa-miR-324-3p*, *hsa-miR-331-3p* and *hsa-miR-451* DNA primers (Exiqon, Denmark) and 2 U of ExiLEN SYBR® Green master mix (Exiqon, Denmark). 20 µl of RT reaction was diluted 20× and 4 µl of the diluted cDNA was used in 10 µl polymerase chain reaction (PCR) amplification reactions. A non-template control (NTC) was added to verify the specificity of the quantitative reverse transcription PCR (qRT-PCR). Reactions of qRT-PCR were carried out using Opticon Monitor 3 (Bio-Rad Laboratories Inc., USA). All reactions were carried out in triplicate. Data of qRT-PCR were assessed according to the 2^-ΔΔCT^ method. All specific amplicons resulted from qRT-PCR was loaded and electrophoresed on 12% non-denaturing polyacrylamide gel electrophoresis (PAGE) in 1X Tris/ Borate/EDTA(TBE) buffer along with 50 bp DNA ladder (Thermo Fisher Scientific, USA) and visualized by silver staining. 

### Statistical analysis

Statistical tests were executed by SPSS (version 21, IBM Corporation, USA). Student’s independent t test was done to analyze the quantitative expression level of *hsa-miR-324-3p* and *hsa-miR-331-3p* between different groups. For all analyses P<0.05 were considered statistically significant.

## Results

### Enrichment and signaling pathways

**Fig.1:**
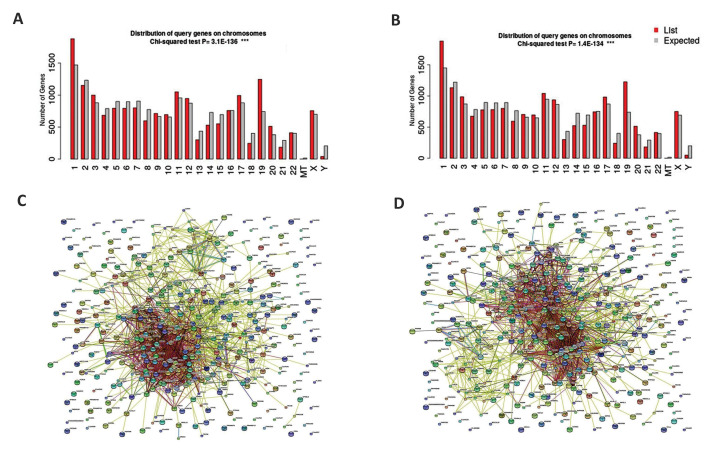
Predicted target genes were totally pertained to *hsa-miR-324-3p* and *hsa-miR-331-3p*, mainly located on the chromosomes 1, 19, 2 and 11 by KEGG server results. **A.** Chromosomal distribution of predicted target genes for *hsa-miR-324-3p*. **B.** Chromosomal distribution of predicted target genes for *hsa-miR-331-3p*. **C.** Network analysis for *hsa-miR-324-3p* by STRING server. **D. **Network analysis for *hsa-miR-331-3p* by STRING server.

**Fig.2:**
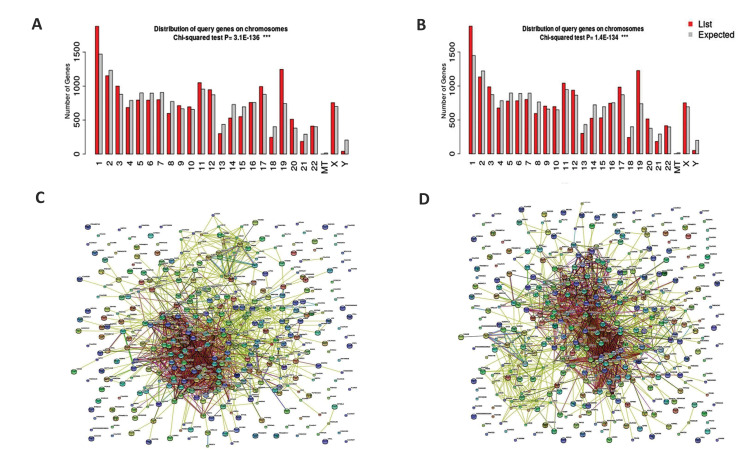
Predicted target genes were totally pertained to *hsa-miR-324-3p* and *hsa-miR-331-3p*, mainly located mainly on the chromosomes 1, 19, 2 and 11 by GO server results. **A.** Chromosomal distribution of predicted target genes for *hsa-miR-324-3p*. **B.** Chromosomal distribution of predicted target genes for *hsa-miR-331-3p*. **C.** Network and protein-protein interaction analysis for *hsa-miR-324-3p* by STRING server. **D. **Network and protein-protein interaction analysis for*hsa-miR-331-3p* by STRING serve.

In the case of predicted targets of *hsa-miR-324-3p* from mirDIP server, it was notable that integrated scores was ranged between 0.067 and 0.014. In this server, predicted targets of *hsa-miR-331-3p* were qualified between 0.74 and 0.014 by integrated scores. In the case of predicted targets of *hsa-miR-324-3p* from TargetScan 7.1, it was noteworthy that total context++ score was between -1.87 and -0.05. In addition, in this server, predicted targets of *hsa-miR-331-3p* were qualified between -0.87 and -0.26 for total context++ score. Using KEGG server, 12656 predicted target genes were totally pertained to *hsa-miR-324-3p* and *hsa-miR-331-3p* (Table S1, S2) (See Supplementary Online Information at www.celljournal.org), mainly located on the chromosomes 1, 19, 2 and 11 by P=3.1E-136 and P=1.4E-134, respectively (Fig .1A, B). Metabolic pathways for *hsa-miR-324-3p* and *hsa-miR-331-3p* were engaged as the top predicted pathways by P= 2.7E-36 and P= 7.7E-37, respectively [Tables S1, S2 (See Supplementary Online Information at www.celljournal.org), Fig.1C, D]. However, using GO Biological process option, 76552 predicted target genes were totally related to *hsa-miR-324-3p* and *hsa-miR-331-3p* (Tables S3, S4, See Supplementary Online Information at www.celljournal.org) mainly located on the chromosomes 1, 19, 2 and 11 by P=3.1E-136 and P=1.4E-134 (Fig .2A, B). Cellular response to stress pathway for *hsa-miR-324-3p* and hsa-miR-331- 3p were engaged, as the top predicted pathways by P= 2.6E-162 and P=5.6E-166, respectively [Tables S3, S4 (See Supplementary Online Information at www. celljournal.org), Fig.2C, D).

### Expression analysis of *hsa-miR-324-3p* and *hsa-miR-331-3p* in Alzheimer’s disease serum samples

**Fig.3:**
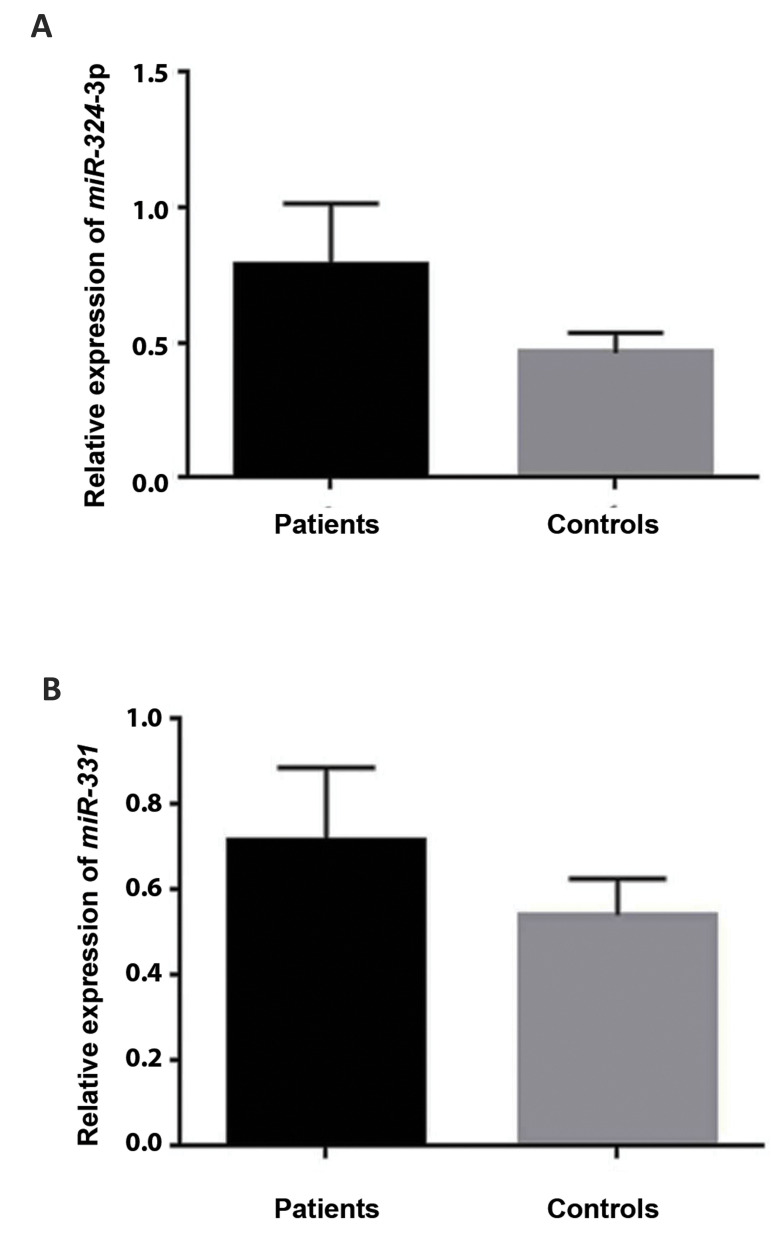
The relative expression graphs of *hsa-miR-324-3p* and *hsa-miR-331-3p*. **A.** *hsa-miR-324-3p* was upregulated in Alzheimer’s disease (AD) patients compared to healthy controls (P=0.61). **B.** *hsa-miR-331-3p* was downregulated in AD patients compared to healthy controls (P=0.78).

Based on the qRT-PCR conclusions, the amplification curve of *hsa-miR-451* with average 21.28 threshold cycle (Ct) and melting curve with 69.8˚Cas temperature (Tm) of primers by single pick and specific amplicon were seen (Table 1). The amplification curve of *hsa-miR-324-3p* and *hsa-miR-331-3p* had 30.92 and 31.77 Ct averages of AD samples in comparison with 32.01 and 34.97 Ct averages of healthy controls, respectively. A 69.9˚Cmelting curve for *hsa-miR-324-3p* and *hsa-miR-331-3p* by single pick and specific amplicon were observed. 12% PAGE system showed one specific amplified product for three miRNAs including *hsa-miR-324-3p*, *hsa-miR-331-3p* and internal control of *hsa-miR-451* (Fig .S1, See Supplementary Online Information at www.celljournal.org). Using GraphPad Prism 7 software, expression level and fold change graph were depicted. Among the studied AD patients, expression level of *hsa-miR-324-3p* andhsa-miR-331- 3p showed upregulation in comparison with the healthy controls (Fig.3A, B). Statistical analyses revealed that upregulated expression of *hsa-miR-324-3p* and *hsa-miR-331-3p* were not statistically significant, by respectively P=0.61 and P=0.78.

**Table 1:** Features of Alzheimer’s disease (AD) patients and healthy controls entered into the study


AD patients	Sex		Age (Y)			Treatment status	
	Female	Male	50-65	65-80	80-95	Under treatment	Without treatment

Percent (%)	71.42	28.57	28.57	47.61	23.80	14.28	85.71
Healthy controls	Female	Male	50-65	65-80	80-95	Lack of special disease history	With Age-related diseases
Percent (%)	43.47	56.52	65.21	30.43	4.34	86.95	13.04


## Discussion

In silico analysis showed *hsa-miR-324-3p* and *hsa-miR-331-3p* are involved in the cellular response to stress and metabolic pathways particularly nucleotide metabolism. It seemed that the functions of these two miRNAs were directly or indirectly associated with AD pathophysiology. Totally, the successful treatment or prevention of AD remains elusive, because the molecular mechanisms driving AD pathology remain poorly understood (34). Different views of mitochondrial dysfunction have been currently characterized as novel components in the aetiology of AD. This is true not only for neuronal mitochondria, but also for astroglial cells which possess strong influence on neuronal function, neuronal development. It has been related to the various neurodegenerative diseases, encompassing AD and other forms of dementia (35, 36). Buffering neurotransmitters and ions (37) and metabolize glucose to lactate, the major fuel for neurons (38), supplying neurons with substrates for oxidative phosphorylation (39, 40) are main mitochondrial functions. On the other hand, damaged mitochondria have been identified in brain tissue, in both familial and sporadic types of AD. In addition, AD is characterized by enhanced numbers of somatic, mitochondrial DNA (mtDNA) mutations, defect of oxidative phosphorylation, imbalance between mitochondrial fission and fusion as well as the alterations in mitochondrial structure, dynamics and motility. Analysis of hippocampal biopsies by microarray have demonstrated a significant reduction of nuclear and one mitochondrial encoded subunits of the mitochondrial electron transport chain in AD patients compared to the age-matched controls. The inverse Warburg hypothesis suggested a bioenergetic model for AD, presuming that AD is an outcome of mitochondrial deregulation, concluding metabolic reprogramming as a beginning attempt to retain neuronal integrity. When this compensatory mechanism is exhausted, bioenergetic deficiencies may result in neuronal death and dementia. Therefore, mitochondrial dysfunction may indicate the missing connection between aging and sporadic AD, andit shows attractive targets against neurodegeneration. Usually, mitochondrial dysfunctions related to AD include direct or indirect prevention of the ability of neuronal and glial mitochondria to carry out oxidative phosphorylation. One direct issue of decreased oxidative phosphorylation is a decline of the ATP production and alterations of mitochondrial bioenergetics crucial for the vitality of cells. An important elevation of oxidized biomolecules has been determined as a sign of AD in brain tissue. Postmortem brains of AD patients showed increased oxidative base damage in both nuclear DNA (nDNA) and mtDNA. In addition, lipid peroxidation of neuronal tissue and oxidative modifications of proteins has been shown in AD. Increased oxidative stress promotes the formation of Aβ plaques and NFTs has been indicated in a mouse model. Oxidative damage is shown to be quantitatively most predominant early event and it is decreased with disease progression in human post-mortem AD brains (7).

The increased levels of reactive oxygen species (ROS) in AD brain has often been proposed by a mitochondrial origin. The high oxygen consumption rate of neurons is used for oxidative phosphorylation and accumulated damaged mitochondria in the AD brain. ROS are endlessly made from up to 4% of the oxygen consumed, during the process of oxidative phosphorylation, by this process. Complex I and especially complex III of the electron transport chain are the initial position for electron leakage to molecular oxygen producing the superoxide anion (·O_2_^-^). Production of ROS is inversely related to the rate of electron transport, elevating exponentially when complex I or III are damaged. Mitochondrial generated ROS as a second messenger molecule suggested to report oxygen available for oxidative phosphorylation, influencing epigenetic marking of nDNA and regulating nuclear transcription factors, kinases and phosphatases.·O_2_^ -^ is neutralized by intramitochondrial Manganese (Mn)-dependent superoxide dismutase (SOD_2_) catalyzing the formation of H_2_ O_2_ , which in turn is inactivated by glutathione peroxidase. If the amount of generated ROS exceeds the capacity of the mitochondrial antioxidant enzymes, ·O_2_^ -^ and H_2_ O_2_ levels will be increased. The highly reactive OH^·^ can be generated by Haber-Weiss or Fenton reactions, in the presence of transition metals, such as iron or copper. OH^·^ accumulation can sequentially lead to a plethora of ROS, which has the potential to induce oxidative harm to lipids, proteins, RNA and DNA. Microglia cells are the origin of increased generation of ROS as a part of an inflammatory response in the AD brain. Supporting the idea, NADPH oxidase, the major mediator of microglial ROS, has been shown to be activated in the AD brain, as assessed by translocation of NADPH oxidase subunits. Current studies propose that mitochondrial generated ROS can work as regulators of pro-inflammatory responses of microglia cells. In case of nucleotide metabolism, nucleotide levels in eukaryotes are retained by nucleotide salvage and/or de novo synthesis of ribonucleotide triphosphate (rNTPs) and deoxyribonucleotide triphosphate (dNTPs). Imbalanced dNTP pools have been shown in AD patients and may be an early risk biomarker for AD. Although the underlying detailed mechanism remains unknown, it is conceivable rNTP and/or dNTP pool imbalances carry out a function in AD aetiology. Oxidative phosphorylation is related to the synthesis of rNTP and dNTP indirectly via production of ATP and directly throughout the enzyme dihydroorotate dehydrogenase (DHODHase). ATP is a main source of cellular bioenergetic process. This molecule is utilized as a substrate for rNTP and dNTP synthesis. Furthermore, binding of ATP to the active site of ribonucleotide reductase (RNR) is essential for the key enzyme activation of the dNTP de novo synthesis. DHODHase is an integral protein of the inner mitochondrial membrane encountering the inter membrane space and involving in the conversion of dihydroorotate to orotate. This product is subsequently converted into uridine monophosphates, pyrimidine deoxyribonucleotides. Functionally, DHODHase is linked to the oxidative phosphorylation by a flavinnon-protein group coupling dihydroorotate oxidation to respiratory ubiquinone reduction. Hypoxemia ribonucleotides and pyrimidine, prevention of oxidative phosphorylation, presence of electron transport chain inhibitors or mutations of complex III and IV of the electron transport chain leads to the deficiencies of the de novo UMP synthesis and a subsequent decline in the de novo synthesis of pyrimidines. 

dNTP levels are significant substrates for mitochondrial DNA replication and post replicative DNA repair processes in post-mitotic cells such as neurons. rNTP levels serve as the substrates for RNA synthesis, pyrimidine ribonucleotides and the precursors for phospholipids, glycolipids and glycoproteins synthesis of the plasma membrane. Supporting the idea, expression of genes encoding essential proteins for de novo synthesis of pyrimidines and DHODHase have been indicated in neuronal cell bodies of rat brain. High expression levels of DHODHase and other de novo components were recognized in the neocortex and hippocampus which were severely influenced in AD patients. The fundamental functions of generated pyrimidines by mitochondria in neurons can suggest a role for the engagement of imbalanced dNTP levels in the aetiology of AD. 

According to the in silico and enrichment analysis, engagement of the nucleotide metabolism and cellular response to stress genes was probably confirmed as the targets of *hsa-miR-324-3p* and *hsa-miR-331-3p*. Experimentally, qRT-PCR results of these two miRNAs were upregulated but not statistically significant. Our result was in accordance with Vallelunga et al. (23) result showing that upregulation of *hsa-miR-324-3p* was occurred in the PD and MSA patient’s serums. In addition, our result was in accordance with Olivieri et al. (25) results indicating upregulation of *hsa-miR-331-3p* in AD serum samples. They explained upregulation of the miRNA in age-related disorders by miRNA array method. They also reported the high expression level of *hsa-miR-331-3p* in AD-plasma samples.

## Conclusion

Based on the results of present study, has-miR-324-3p and *hsa-miR-331-3p* expression levels did not significantly increase in the patients suffering Alzheimer. It may be concluded that these two miRNAs are not involved in the pathogenesis of AD.

## Supplementary PDF


