# Supplementary material for: Identification of Circulating hsa-miR-324-3p and hsa-miR-331-3p Exchanges in The Serum of Alzheimer’s Patients and Insights into The Pathophysiological Pathways
Source: Cell J. 2021 May 26;23(2):211–7. doi: 10.22074/cellj.2021.7047 (PMC8181312; doi:10.22074/cellj.2021.7047)
Supplement: Supplementary file 1 [file Cell-J-23-211-s01.pdf]

CELL JOURNAL (Yakhteh)

Supplementary Information for

Identification of Circulating *hsa-miR-324-3p* and *hsa-miR-331-3p* Exchanges in The Serum of Alzheimer's Patients and Insights into The Pathophysiological Pathways

Maryam Heydari, M.Sc., Zohreh Hojati, Ph.D.*, Moein Dehbashi, Ph.D.

Division of Genetics, Department of Cell and Molecular Biology and Microbiology, Faculty of Biological Science and Technology, University of Isfahan, Isfahan, Iran

**Corresponding Address: P.O.Box: 81746-73441, Division of Genetics, Department of Cell and Molecular Biology and Microbiology, Faculty of Biological Science and Technology, University of Isfahan, Isfahan, Iran*
Email: z.hojati@sci.ui.ac.ir

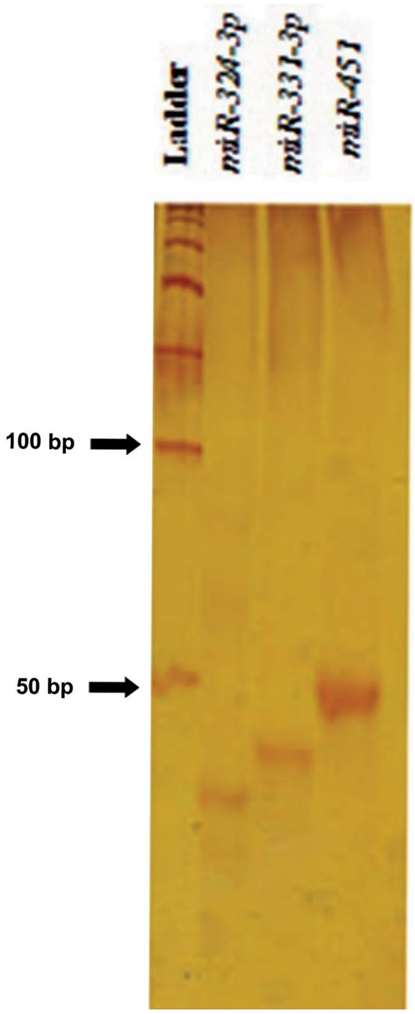

Fig.S1: Non-denaturing PAGE was applied for electrophoresis of qRT-PCR products. Left to right lanes: Ladder 50 bp, *hsa-miR-324-3p, hsa-miR-331-3p* and *hsa-miR-451*. PAGE; Polyacrylamide gelelectrophoresis and qRT-PCR; Quantitative reverse transcription polymerase chain reaction.

Table S1: KEGG server results for enrichment analysis of *hsa-miR-324-3p*

**Adjusted P value**	**Number of genes**	**Pathways**
0.00E+00	1086	Metabolic pathways
4.50E-156	308	Pathways in cancer
5.00E-153	317	PI3K-Akt signaling pathway
8.90E-116	238	MAPK signaling pathway
1.40E-108	245	Neuroactive ligand-receptor interaction
5.40E-103	197	Focal adhesion
1.10E-99	233	Cytokine-cytokine receptor interaction
2.00E-99	206	Proteoglycans in cancer
4.70E-99	199	Regulation of actin cytoskeleton
7.20E-97	195	Rap1 signaling pathway
1.00E-93	203	Rassignaling pathway
6.40E-92	221	HTLV-I infection
1.50E-91	171	Calcium signaling pathway
3.30E-82	178	Endocytosis
2.30E-81	158	Purine metabolism
3.40E-77	155	Protein processing in endoplasmic reticulum
3.60E-77	148	Oxytocin signaling pathway
3.10E-76	151	cGMP-PKG signaling pathway
5.80E-75	155	Transcriptional misregulation in cancer
7.40E-74	142	Hippo signaling pathway
1.90E-69	167	Epstein-Barr virus infection
4.10E-69	131	Ubiquitin mediated proteolysis
4.40E-69	160	Chemokine signaling pathway
5.60E-68	161	Huntington’s disease
5.60E-68	161	Viral carcinogenesis
1.70E-67	135	Adrenergic signaling in cardiomyocytes
4.00E-66	128	Insulin signaling pathway
5.70E-66	130	Wntsignaling pathway
1.30E-63	134	MicroRNAs in cancer
4.70E-63	120	Axon guidance
-	6333	Total

Table S2: KEGG server results for enrichment analysis of *hsa-miR-331-3p*

**Adjusted P value**	**Number of genes**	**Pathways**
0.00E+00	1079	Metabolic pathways
1.30E-156	319	PI3K-Akt signaling pathway
1.30E-156	308	Pathways in cancer
9.40E-118	239	MAPK signaling pathway
3.40E-103	241	Neuroactive ligand-receptor interaction
9.70E-103	201	Regulation of actin cytoskeleton
2.40E-101	234	Cytokine-cytokine receptor interaction
2.40E-101	207	Proteoglycans in cancer
1.20E-100	197	Rap1 signaling pathway
1.40E-99	195	Focal adhesion
1.10E-98	206	Rassignaling pathway
3.70E-92	221	HTLV-I infection
1.20E-89	170	Calcium signaling pathway
2.20E-79	176	Endocytosis
4.00E-79	156	Protein processing in endoplasmic reticulum
4.70E-75	155	Transcriptional misregulation in cancer
1.30E-74	150	cGMP-PKG signaling pathway
5.70E-74	142	Hippo signaling pathway
1.20E-73	146	Oxytocin signaling pathway
5.70E-73	153	Purine metabolism
3.10E-69	131	Ubiquitin mediated proteolysis
4.30E-68	161	Viral carcinogenesis
1.40E-67	135	Adrenergic signaling in cardiomyocytes
4.40E-67	165	Epstein-Barr virus infection
1.60E-65	159	Huntington’s disease
2.10E-64	127	Insulin signaling pathway
5.40E-64	156	Chemokine signaling pathway
9.30E-64	134	MicroRNAs in cancer
3.60E-63	120	Axon guidance
3.30E-62	140	Jak-STAT signaling pathway
-	6323	Total

Table S3: GO server results for enrichment analysis of *hsa-miR-324-3p*

**Adjusted P value**	**Number of genes**	**Pathways**
2.60E-162	1436	cellular response to stress
2.70E-156	1389	positive regulation of cell communication
2.90E-151	1392	regulation of protein modification process
5.30E-149	1406	positive regulation of macromolecule biosynthetic process
1.90E-145	1405	cell development
1.20E-140	1272	positive regulation of signaling
1.10E-138	1240	response to endogenous stimulus
1.50E-138	1352	tissue development
2.90E-138	1224	regulation of intracellular signal transduction
4.70E-137	1295	positive regulation of RNA metabolic process
8.20E-137	1328	regulation of phosphorus metabolic process
6.50E-136	1316	regulation of phosphate metabolic process
6.90E-133	1156	positive regulation of signal transduction
4.40E-132	1261	positive regulation of RNA biosynthetic process
1.40E-130	1330	regulation of multicellular organismal development
7.40E-130	1384	organonitrogen compound metabolic process
3.70E-129	1281	regulation of cell proliferation
7.80E-129	1221	positive regulation of protein metabolic process
8.50E-129	1238	positive regulation of transcription, DNA-templated
8.50E-129	1238	positive regulation of nucleic acid-templated transcription
3.40E-128	1228	neurogenesis
5.20E-128	1244	positive regulation of catalytic activity
1.20E-123	1168	generation of neurons
1.80E-122	1142	positive regulation of cellular protein metabolic process
4.00E-122	1308	organic substance catabolic process
3.20E-121	1275	regulation of cell death
1.40E-120	1117	regulation of phosphorylation
9.30E-120	1270	regulation of cell differentiation
9.20E-119	1175	positive regulation of multicellular organismal process
4.20E-118	1159	response to oxygen-containing compound
-	38250	Total

Table S4: GO server results for enrichment analysis of *hsa-miR-331-3p*

**Adjusted P value**	**Number of genes**	**Pathways**
5.60E-166	1437	cellular response to stress
2.80E-154	1385	positive regulation of cell communication
2.10E-153	1408	positive regulation of macromolecule biosynthetic process
1.20E-151	1390	regulation of protein modification process
7.90E-147	1404	cell development
1.70E-142	1226	regulation of intracellular signal transduction
3.20E-141	1297	positive regulation of RNA metabolic process
1.00E-139	1269	positive regulation of signaling
1.00E-138	1238	response to endogenous stimulus
5.80E-138	1349	tissue development
2.90E-137	1264	positive regulation of RNA biosynthetic process
4.20E-137	1326	regulation of phosphorus metabolic process
3.60E-136	1314	regulation of phosphate metabolic process
1.10E-135	1333	regulation of multicellular organismal development
5.70E-135	1242	positive regulation of transcription, DNA-templated
5.70E-135	1242	positive regulation of nucleic acid-templated transcription
7.20E-134	1155	positive regulation of signal transduction
2.40E-133	1231	neurogenesis
2.10E-130	1382	organonitrogen compound metabolic process
9.00E-129	1171	generation of neurons
6.90E-126	1216	positive regulation of protein metabolic process
1.50E-124	1273	regulation of cell differentiation
3.30E-124	1238	positive regulation of catalytic activity
1.20E-121	1176	positive regulation of multicellular organismal process
2.10E-121	1116	regulation of phosphorylation
4.70E-121	1270	regulation of cell proliferation
1.00E-120	1304	organic substance catabolic process
2.70E-119	1137	positive regulation of cellular protein metabolic process
2.90E-116	1242	cellular catabolic process
3.60E-116	1267	regulation of cell death
-	38302	Total
